# Role of epigenetic modification in interferon treatment of hepatitis B virus infection

**DOI:** 10.3389/fimmu.2022.1018053

**Published:** 2022-10-17

**Authors:** Zhijing Yang, Baozhen Sun, Jingcheng Xiang, Han Wu, Shaoning Kan, Ming Hao, Lu Chang, Huimin Liu, Dongxu Wang, Weiwei Liu

**Affiliations:** ^1^ Department of Oral and Maxillofacial Surgery, Hospital of Stomatology, Jilin University, Changchun, China; ^2^ Department of Hepatobiliary and Pancreas Surgery, China-Japan Union Hospital of Jilin University, Changchun, China; ^3^ Jilin Provincial Key Laboratory of Tooth Development and Bone Remodeling, Hospital of Stomatology, Jilin University, Changchun, China; ^4^ Laboratory Animal Center, College of Animal Science, Jilin University, Changchun, China

**Keywords:** HBV, IFN therapy, ISGs, epigenetic regulation, CccDNA

## Abstract

Human hepatitis B virus (HBV) is a small, enveloped DNA virus that causes acute and chronic hepatitis. Chronic hepatitis B (CHB) is associated with hepatocellular carcinoma pathogenesis. Interferons (IFNs) have been used for the treatment of CHB for a long time, with advantages including less treatment duration and sustained virological response. Presently, various evidence suggests that epigenetic modification of the viral covalently closed circular DNA (cccDNA) and the host genome is crucial for the regulation of viral activity. This modification includes histone acetylation, DNA methylation, N6-methyladenosine, and non-coding RNA modification. IFN treatment for CHB can stimulate multiple IFN-stimulated genes for inhibiting virus replication. IFNs can also affect the HBV life cycle through epigenetic modulation. In this review, we summarized the different mechanisms through which IFN-α inhibits HBV replication, including epigenetic regulation. Moreover, the mechanisms underlying IFN activity are discussed, which indicated its potential as a novel treatment for CHB. It is proposed that epigenetic changes such as histone acetylation, DNA methylation, m6A methylation could be the targets of IFN, which may offer a novel approach to HBV treatment.

## Introduction

Hepatitis B is a global epidemic that remains a great challenge (1, 2). After acute infection with the hepatitis B virus (HBV), 90%–95% of patients can be cured completely; however, the remaining 5%–10% can act as carriers, allowing chronic HBV infections to spread within populations (3). Moreover, HBV infection leads to liver cirrhosis and hepatocellular carcinoma (HCC), which can be fatal (4, 5). China is the country with the heaviest burden of HBV infection and liver cancer is the second most common cancer in China (6, 7).

**Figure 1:**
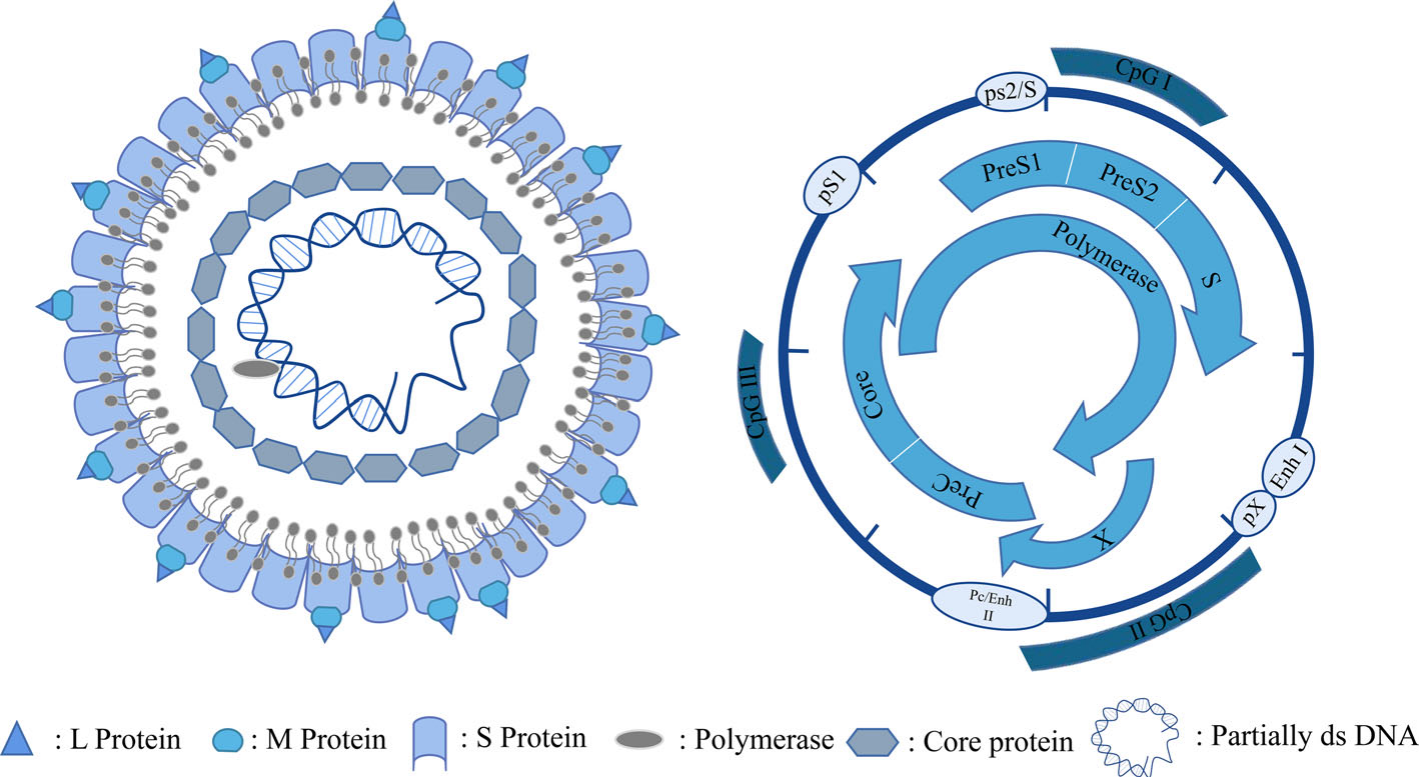
HBV particles and genome.

HBV is a small DNA virus belonging to the family Hepadnaviridae. Its genetic material is 3.2 kb long and consists of relaxed, circular, partially double-stranded DNA, which is enclosed within the nucleocapsid core of the virus (8). HBV enters hepatocytes through a receptor-mediated pathway and binds to the receptor sodium taurocholate cotransporter polypeptide (NTCP) on the surface of hepatocytes *via* its envelope proteins PreS1 and PreS2 (9). After entering the hepatocytes, the relaxed, circular, partially double-stranded DNA (rcDNA) of HBV is released from the viral nucleocapsid and transported into the nucleus (10). Redundant sequences at the pol-linked terminal on minus strand DNA and RNA oligonucleotides at the 5’ ends of plus strand DNA are removed from rcDNA and gaps on both strands are filled in and connected to generate cccDNA (11–13). CccDNA is used as a template for the transcription of pregenomic RNA (pgRNA) and subgenomic mRNA (**Figure 1**) (14, 15). PgRNA was catalyzed by HBV polymerase to synthesize viral genomic DNA. Meanwhile, subgenomic mRNA is translated into various viral proteins as a part of the HBV life cycle. Among the proteins, the packaging proteins are packaged with the nascent viral DNA to form progeny virus and released from the cells (16). HBV cccDNA is important for chronic infection (17) and exists in the nucleus as a minichromosome, bound to histones and non-histone proteins. The epigenetic modification of cccDNA contributes to the viral replication and affects the prognosis of chronic HBV infection (18). Therefore, epigenetic modifications, such as DNA methylation, RNA methylation, histone acetylation, miRNA regulation, and chromatin remodeling, can regulate the activity of cccDNA and offer new therapeutic targets for HBV infection.

Current Food and Drug Administration (FDA)-approved treatments for chronic HBV infection include interferon-α (IFN-α) and nucleoside analogs (such as lamivudine, adefovir dipivoxil, entecavir, telbivudine, and tenofovir fumarate dipivoxil) (19). IFN-α has various therapeutic advantages, which include less treatment time and higher clearance of hepatitis B antigen and surface antigen (20). Moreover, IFN-α has both immunomodulatory and antiviral effects (21) and can inhibit cccDNA activity through epigenetic repression (22). In this review, we have discussed the mechanism underlying the IFN treatment for HBV infection and described the epigenetic modification of HBV through IFN treatment.

## IFN treatment for hepatitis B virus infection

**Figure 2:**
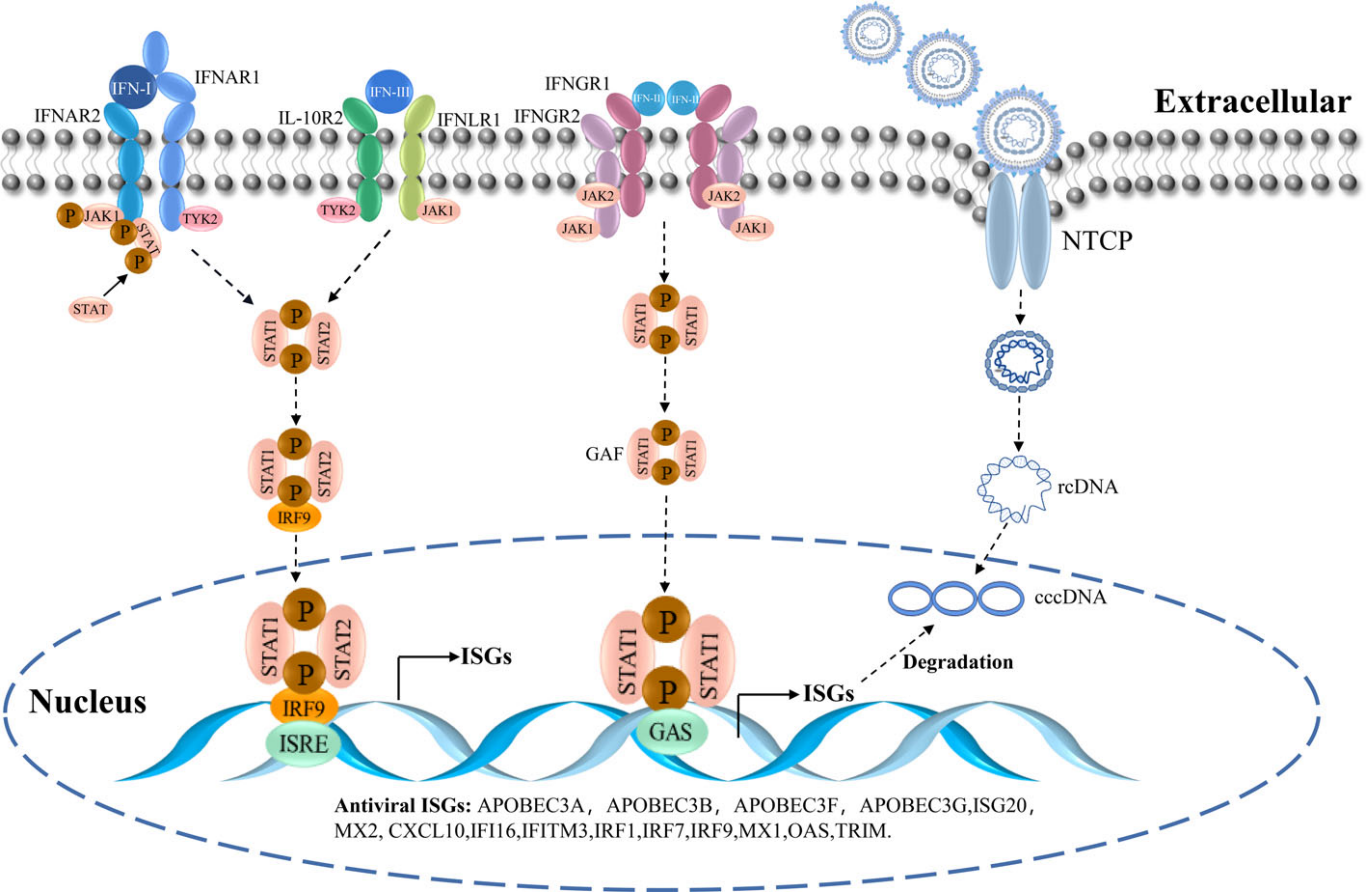
The IFN-signaling cascade and the entry of HBV.

IFN is a natural immune substance with broad-spectrum antiviral activity (23). It is released by virus-infected cells. The following three types of IFNs have been identified: type I IFN (including IFN-α, -β, -ϵ, -κ, -ω, and others), type II IFN (IFN-γ), and type III IFN (IFN-λ) (24). IFN inhibits virus replication by promoting the expression of downstream IFN-stimulated genes (ISGs) *via* multiple signaling pathways (25, 26). Different types of IFNs bind to distinct receptors on the surfaces of cognate cells. IFN-I binds to IFN-α receptor 1 (IFNAR1) and 2 (IFNAR2) heterodimers, whereas IFN-III binds to interleukin-10 receptor 2 (IL-10R2) and IFN-λ receptor 1 (IFNLR1) heterodimers, and IFN-II binds to heterodimers consisting of IFN-γ receptor 1 (IFNGR1) and 2 (IFNGR2) (27). The binding of IFNs to their receptors initiates the signaling cascades *via* the Janus kinase (JAK)- signal transducer and activator of transcription (STAT) pathway, leading to the transcriptional regulation of several genes (28). The binding of IFN-I and IFN-III to their respective receptors leads to the phosphorylation of TYK-2 near JAK1, which is followed by further activation of STAT1 and STAT2. Phosphorylated STAT1 and STAT2 bind to IRF-9, which forms the heterotrimeric complex ISGF3 (29). Subsequently, ISGF3 is transported into the nucleus where it binds to ISREs and activates ISG transcription. The intracellular domains of the IFN-II receptors, IFNGR1 and IFNGR2, bind and activate JAK1 and JAK2 kinases, respectively. They induce phosphorylation of STAT1 and STAT2, which subsequently form homodimeric GAF. This is transported into the nucleus where it binds to GAS and induces ISG transcription (30–32) (**Figure 2**).

ISGs play an important role in host resistance to HBV infection. It inhibits viral entry and exit as well as viral replication, transcription, translation, and post-translational modification (33, 34). For the invasive phase of the virus, ISGs include the myxovirus resistance gene (Mx) and the IFN -induced transmembrane proteins CH25H, viperin, and tetherin. IFN-induced proteins are capable of inhibiting viral replication and transcription. They include 2′-5′-oligoadenylate synthetase (OAS), protein kinase R (PKR), ZAP, IFN-induced protein with tetratricopeptide repeats, ISG15, and members of the TRIM family. Furthermore, the release phase of the virus is inhibited by proteins, including viperin and tetherin (35, 36). The SMAD4A can bind to the Smaug recognition region (SRE) sequence in the HBV virus sequence to trigger the degradation of the virus (37). TRIM25 is downregulated in HBV patients, and TRIM25 overexpression results in increased IFN production and decreased HBV replication (38). The proteomic data analysis showed that after IFN treatment, the levels of the proteins RIG-I and RIG-G (known to be suppressed by HBV) are restored. Moreover, RNA metabolism, translation, and endoplasmic reticulum targeting are differentially regulated in the biological process (39). Therefore, as a class of effector molecules mediating antiviral effects, ISGs can target virtually all the processes of viral invasion, uncoating, genome replication, and virion assembly and release, thereby inhibiting viral proliferation *in vivo* (**Figure 2**).

### Clinical applications of IFN in the treatment of HBV

Presently, seven therapies have been approved by the FDA for their use in HBV clinical treatment, including standard IFN and pegylated interferon (peg-IFN) (19). Peg-IFN has been recommended by the guidelines of the major liver associations (40, 41). The course of peg-IFN therapy is finite and can lead to long-term benefits, such as continuous and cumulative responses. Moreover, the progression of hepatitis to fibrosclerosis and hepatocellular carcinoma can be reduced (42). The clinical applications of interferon and its mechanisms underlying the HBV treatment are discussed below.

Even though the mechanisms underlying IFN treatment for hepatitis B are still being elucidated, IFN has been used in the clinical treatment of HBV for decades and remains an effective therapy. For patients with chronic hepatitis B (CHB), standard (peg-IFN) monotherapy is administered subcutaneously once weekly for 48 weeks, which has decreased treatment time and sustained virological response (43). After peg-IFN-α treatment, some patients with CHB maintained a functional cure, related to lower HBcrAg and higher HBsAb levels (44). Clinical trial results have shown that peg-IFN-α2b treatment can lead to a greater decrease in HBV DNA in patients with HBeAg positive compared with patients who received an only placebo (45). In addition, peg-IFN-α2b was effective in approximately one-third of patients who were refractory to standard IFN or lamivudine therapy (46). Compared with HBeAg-negative patients who received Entecavir (ETV) monotherapy, HBeAg-negative patients who received peg-IFN monotherapy showed a significantly greater decline in HBsAg levels (47).

IFN can also be used as an adjuvant with other types of drugs to achieve better HBV treatment effects. The peg-IFN-nucleoside analog (NA) sequential optimization therapy (SOT) can lower HBsAg, undetectable HBV DNA, and ALT normalization compared with peg-IFN monotherapy (48). One study showed that after 48 weeks of treatment, HBsAg in the early combined treatment group (ETV plus Peg-IFN-α-2a) decreased by more than 1500 IU/mL, and the average HBsAg level was significantly lower than that in the late combined treatment group and the NA monotherapy group (P <0.05) (49). In another study, after peg-IFN treatment, responses were doubled in patients that were not treated with peg-IFN with HBsAg below 4000 IU/mL and HBV DNA below 50 IU/mL. These patients are the candidates for peg-IFN add-on therapy (50). In addition, the combination therapy after hepatitis B vaccination significantly increased the seroclearance of HBsAg (51).

Presently, HBV cannot be completely cured by current approved clinical drugs. Reducing the loss of serum HBV DNA and HBsAg is presently a method that can reduce the probability of transformation to hepatitis and hepatic cell carcinoma. IFN therapy has the advantages of a shorter treatment cycle and fewer treatment times that can improve the patient’s compliance and treatment effectiveness. Moreover, IFN therapy has advantages over NAs in immunology mechanisms and can alter the state of immune tolerance. However, despite the side effects of IFN therapy, the relatively low response rates are a problem. NAs do not target the cccDNA; therefore, the HBV can reactivate. Therefore, combination therapy can exert a better effect on the treatment of CHB.

### The therapeutic mechanism of IFN

IFN can develop direct anti-viral effects and develop a complex immune response in the treatment of HBV. IFN-α14 can suppress the transcription of cccDNA and the production of HBsAg and HBeAg (52). IFN has been shown to increase T cell survival, the expression of T cell antigens, and IL 12 (53). It can induce antiviral activity in hepatocytes through the regulation of gene expression and protein translation, thereby playing a non-cytolytic antiviral role in many stages of the HBV life cycle (54). For example, the MX2 induced by IFN-α can decrease the number of cccDNA and the levels of HBV RNA (55). Furthermore, TRIM14 is an important molecule in the IFN signaling pathway, playing a crucial role in HBV suppression mediated by IFN-I. The TRIM14 SPRY domain interacts with the C-terminus of HBx, which can block the role of HBx in promoting HBV replication by inhibiting the formation of the Smc-HBx-DDB1 complex (56). To elucidate the mechanism underlying IFN treatment in HBV, high-throughput bimolecular fluorescence complementation screening was performed to determine the interactions between HBx protein and 145 ISGs. The results showed that seven HBx-interacting ISGs (GBP2, PVRL4, CBFβ, TRIM38, TRIM5γ, TRIM25, and Gadd45γ) exerted strong inhibitory effects on HBV replication. Among them, TRIM5γ and TRIM31 promoted HBx degradation, which can offer a novel therapeutic method in cases of IFN drug resistance (57). Under the action of a key nuclease for IFN-triggered cccDNA clearance, IFN-α induced the non-cytolytic removal of HBV cccDNA from the nucleus of infected hepatocytes in an A3A deaminase-dependent manner (22, 58). Further cell-based assays were performed to detect the anti-viral activity of ISGs. These results showed that SMAD4A was the strongest suppressor of HBV replication, functioning through an SRE-like sequence located in viral RNA. Moreover, the SMAD family inhibited HBV in mouse models (37). Elucidating the mechanism of action of IFN in the treatment of HBV can contribute to the development of IFN-related drugs in clinical practice and provide new targets for HBV treatment.

## Epigenetic regulation of HBV by IFN

The cccDNA is a major hindrance in the treatment of HBV infection (12, 59). After HBV treatment, cccDNA molecules persist in low numbers, which causes CHB. Epigenetic modification regulates the transcriptional activity of cccDNA chromosomes (60). Therefore, studying the epigenetic modification of cccDNA can aid the development of new therapeutic drugs to eliminate cccDNA (61).

### Epigenetic changes involved in the process of HBV infection

HBV has a tightly condensed DNA genome of only 3.2 kb in length. The four functional viral proteins are produced in overlapping open reading frames (ORFs), which are orientated in the same direction and encoded by negative chains (62), which are as follows: (i) preS/S, encoding the three viral surface proteins (HBs) (termed the small [S], medium [M], and large [L] surface antigens) that bind to the viral envelope and mediate viral entry; (ii) Precore/core, encoding the HBV core protein (HBc) and the secreted e-antigen (HBeAg); (iii) viral polymerase POL, which participates in viral replication and packaging; and (iv) the X region, encoding the X protein (HBx), which has been proved to have multiple functions, such as viral replication, and is the main cause of liver cancer caused by HBV (63, 64). In addition, two enhancers (enhancers I and II) and three predicted CpG islands constitute the structure of HBV cccDNA. HBV cccDNA exists as a minichromosome and undergoes post-translational modification (PTM) of the bound histones (65). Furthermore, the transcriptional activity of cccDNA is highly affected by epigenetic modification (15). IFN and NA treatment have limited treatment effects because they cannot eliminate HBV cccDNA. Therefore, the understanding of epigenetic modifications in the replication and disease development of HBV is important for degrading cccDNA and developing new therapeutic approaches. The epigenetic changes involved in the process of HBV infection are mentioned below.

DNA methylation typically occurs on CpG dinucleotides through the activity of DNA methyltransferases (DNMTs) and is often associated with gene transcriptional silencing (66). The HBc maintains its binding to cccDNA by binding to CpG island 2 in the HBV genome, and its relative abundance is highly associated with the serum HBV DNA levels in patients (67, 68). One study showed that patients with occult hepatitis B infection had a high methylation density at CpG island 2 (69). CpG island 2 methylation was significantly higher in patients with HBeAg-negative. In addition, HBV DNA methylation was higher in CpG islands 2 and 3 in HCC tissues compared with infected and cirrhotic tissues (70–72). HBx expression increased total DNMT activity by upregulating DNMT1, DNMT3A1, and DNMT3A2 and selectively promoted the regional hypermethylation of specific tumor suppressor genes, which caused hepatocarcinogenesis (73). HBV infection cause hypermethylation of the promoter region of the tumor suppressor E-cadherin, the expression of which is frequently absent in HCC (74). A study showed that the HBV DNA demethylation and increased abundance of 5hmc residues in viral CpG sequences is associated with the HBV replication (75). In general, these reports indicated that the DNA modification plays an important role in the HBV replication and HCC development, which may provide a new target for HBV treatment.

Histone acetylation also occurs during the process of HBV infection. Many histone modifying enzymes, such as acetyltransferases (HATs), deacetylases (HDACs), lysine methyltransferases, and protein arginine methyltransferases, can modify histones associated with cccDNA (76). Transcription of HBV minichromosomes is regulated by epigenetic changes in cccDNA-bound histones, whereas the acetylation status of H3/H4 histones that regulate cccDNA binding affects HBV replication (77). Chromatin immunoprecipitation (ChIP)-sequencing assays of HBV cccDNA showed non-randomly distributed PTMs, which strongly suggested that PTMs in the stained cccDNA are specifically introduced after histone assembly. High levels of H3K4me3, H3K27ac, and H3K122ac have been detected in infected cells, which indicates their importance for HBV transcription (78). Therefore, understanding the role of histone acetylation in cccDNA transcription is important for HBV treatment.

While DNA methylation and histone acetylation are targeted to DNA and proteins, RNA transcriptional regulation is also involved in epigenetic modifications in HBV. N6-methyladenosine (m6A) modification is the most common form of modification in eukaryotic cells and viruses, which includes HBV, regulating RNA transcription, splicing, degradation, and translation without changing the base sequence (79, 80). m6A regulates HBV RNA stability and reverse transcription during the HBV life cycle (81). HBV can affect m6A levels in host cells to achieve HBV-directed immune evasion. A study has shown that HBV significantly increased m6A modification of PTEN RNA, which resulted in decreased levels of PTEN protein. In the absence of PTEN, IRF-3 dephosphorylation at Ser97 was diminished and IFN synthesis was impaired (82). Moreover, ChIP analysis of wild-type HBV and HBx-null virus-infected primary human hepatocytes and HepG2-NTCP cells revealed recruitment of METTL3/14 proteins by HBx at transcriptional initiation sites, which led to internal RNA m6A modification (83). Overall, m6A modification occurring during HBV replication directly affected co-transcriptional synthesis and modification of viral RNA.

MiRNA is an important factor in HBV. Cellular miRNAs are specific noncoding RNA molecules and 21–25 nucleotides in length. They regulate gene expression by preventing translation or accelerating RNA degradation by targeting specific mRNAs (84). Viruses can also encode miRNAs that regulate viral replication by altering host gene expression (85). miRNAs can affect HBV replication directly by binding to HBV transcripts or indirectly by targeting cytokines involved in HBV replication (86). HBV-miR-3, an HBV-encoded miRNA whose expression is highly related to HBV activity, promotes the anti-HBV effects of IFN and also induces the production of IL-6 in M1 macrophages (87). Liver tissue samples from 52 of 87 patients with CHB showed expression of HBV-miR-6. The levels of HBV-mir-6 correlate with hepatic HBV DNA and plasma HBsAg levels, suggesting that this molecule can participate in viral excretion or particle formation (88). Therefore, miRNA encoded by HBV can be a new target for HBV treatment.

To summarize, epigenetic modifications involved in the HBV life cycle include DNA methylation, histone acetylation, m6A modification, and miRNA expression. Therefore, a deeper investigation into epigenetic regulatory processes can provide new avenues for controlling CHB.

### The role of epigenetic modification induced by IFN in HBV infection

**Figure 3:**
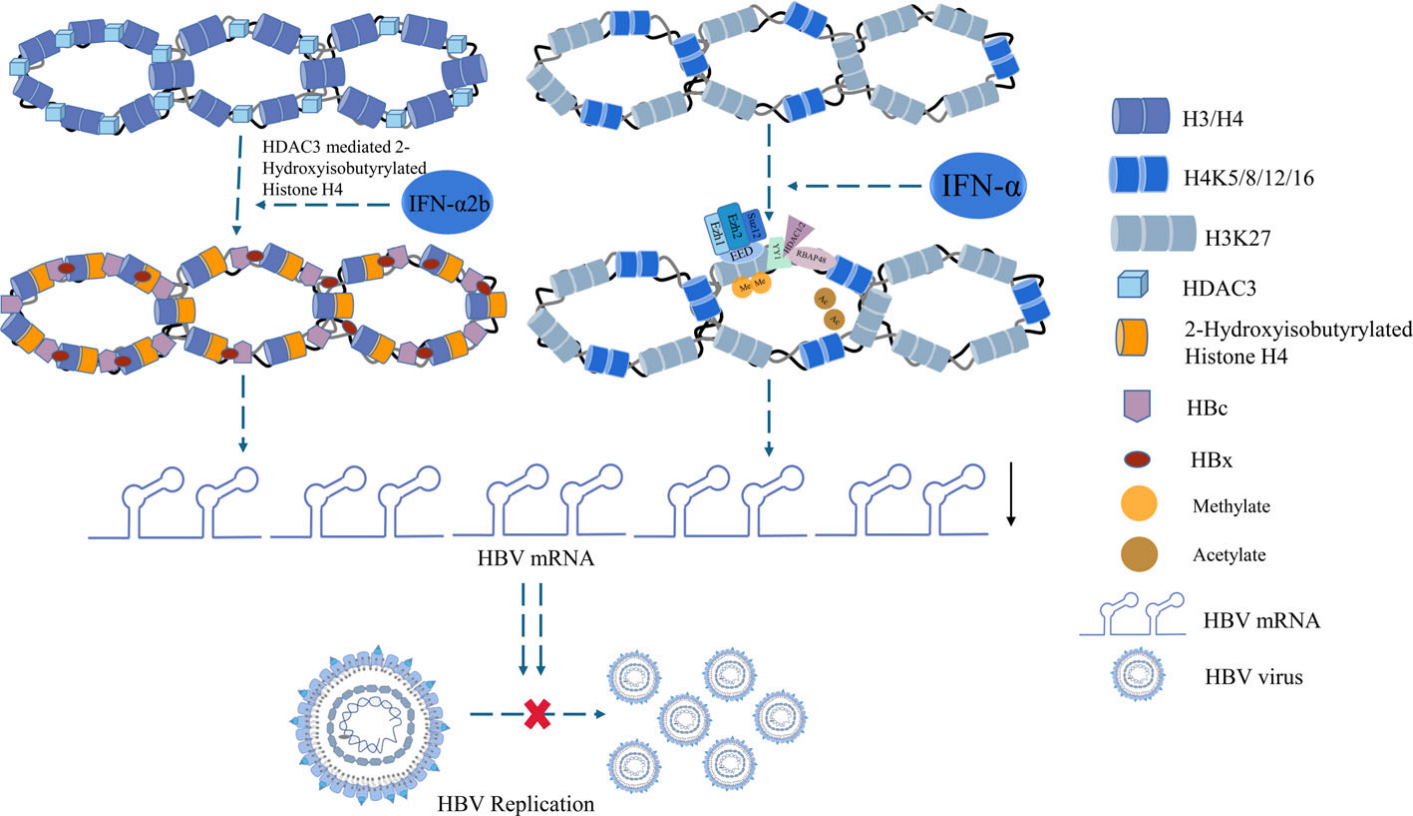
Histone acetylation involved in IFN treatment.

Epigenetic repression of HBV by IFN-α is one of the most important mechanisms of modulating cccDNA modulation (89). IFN-α can regulate the HBV cccDNA minichromosome by modulation of GCN5-mediated succinylation of histone H3K79, facilitating clearance of HBV cccDNA (90). IFN-α2b reportedly increased the HDAC3-mediated de-2-hydroxyisobutyrylation of histone H4 lysine 8 (H4K8) on the HBV cccDNA minichromosome, restricting transcription of the cccDNA in liver (91). Another study showed that the proteins YY1 and Ezh2 (components of the transcriptional repressor complex PRC2) and the histone deacetylases HDAC1 and hSirt1 were actively recruited to cccDNA after IFN-α treatment, which led to the hypoacetylation of cccDNA-bound histones (89) (**Figure 3**). Furthermore, the high-dose IFN-α-mediated upregulation of cytidine deaminase or NFκB pathway induction by antibody-mediated activation of the lymphotoxin-β receptor (LTβR) can promote partial cccDNA degradation (92).

**Figure 4:**
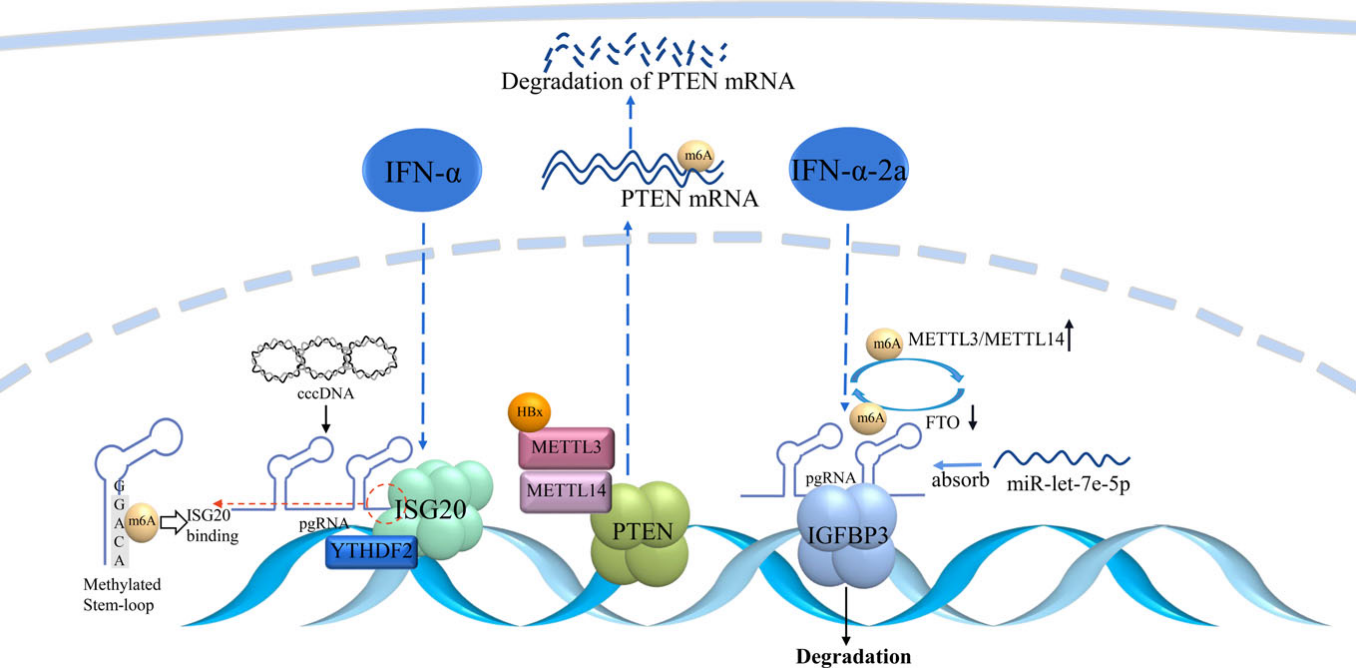
m6A modulation involved in IFN treatment.

Further anti−HBV effects of IFN−α involve m6A modification (**Figure 4**). For example, increased m6A modification of pgRNA was observed after IFN-α2a treatment. The expression of METTL3 and METTL14 was significantly upregulated, whereas that of FTO was downregulated in IFN-α2a-treated HepG2.2.15 cells. These results suggested that IFN-α2a can regulate m6A RNA modification (93). IFN-α can induce ISG20 to selectively degrade m6A-containing HBV RNA. ISG20 can form a complex with YTHDF2 to recruit ISG20, which leads to the degradation of HBV transcripts (94). Overall, IFNs can epigenetically regulate RNA transcription, which leads to transcriptional repression of cccDNA.

Moreover, a study showed that during the peg-IFN treatment, the serum mir-6126 is high, which predicted HBsAg 1-log drop, and the mir-6126 can inhibit HBsAg production and HBV replication *in vitro* (95). A lentivirus-mediated RNAi high-throughput screen for epigenetic modifiers was performed, and it was found that the methyltransferase SETD2 was a key factor involved in the IFN-α mediated antiviral response. It can directly mediate STAT1 methylation on lysine 525 through its methyltransferase activity to increase IFN-α activated STAT1 phosphorylation and antiviral cellular responses (96). IFN can exert the anti-viral effect which inhibits the cccDNA transcription and improve its anti-viral activity through epigenetic modification. The study of epigenetic modification in IFN treatment in HBV infection can offer new insight into the combination of epigenetic drugs and IFN for CHB treatment.

## Discussion

Disease caused by HBV is a global health burden, with 256 million people chronically afected (97, 98). HBV is an enveloped DNA virus, belonging to the Hepadnaviridae family (99, 100). After the entry of the virus into the nucleus, cccDNA is formed through the action of DNA polymerase (101), acting as a stable template for viral transcription (102). Even when antigen levels fulfill the treatment criteria, cccDNA remains in the infected cells and tissues, which can reactivate again (103). Owing to the inherent stability of cccDNA, it is now recognized as the main marker of virus persistence and the main obstacle in curing CHB. IFN therapy is a common therapy used for the treatment of CHB based on its immune response; however, drug resistance is a problem. IFN-α significantly induces IL-6 expression in HBV replicated hepatocytes, which upregulates the expression of cytokine signal transduction inhibitor-3 (SOCS3) and downregulates the expression of downstream effectors of IFN-α. This impairs IFN-α anti-HBV efficiency and leads to IFN resistance (104). Moreover, IFN therapy leads to side effects such as fatigue, thyroid dysfunction, depression, and cognitive slowing because of off-target effects (105). Therefore, IFN in combination with epigenetic modification inhibitors may decrease the off-target effect to achieve a better therapeutic effect.

Because the function of cccDNA is dependent on its epigenetic modification, silencing the activity of cccDNA through epigenetic mechanisms can be an efficient and practical approach to control CHB (18, 106). Epigenetic drugs have been used for the treatment of HBV to silence the transcription of cccDNA. The HAT inhibitor C646 can downregulate H3K27ac and H3K122ac, thereby silencing cccDNA and decreasing HBV transcription (78). Moreover, Gs5801 (Gilead), a prodrug that specifically inhibits lysine demethylase 5, can inhibit cccDNA transcription by removing H3K4me3 (107). SAM can decrease the methylation level of STAT1, which can increase the antiviral effect of IFN-α (108). Because IFN can modulate the epigenetic status of cccDNA, a combination of epigenetic drugs and IFN therapy can be more effective for patients with CHB in the future. To develop such therapies, further elucidation of the molecular mechanisms underlying cccDNA epigenetic regulation in IFN treatment is required.

## Conclusion

IFN is crucial to HBV treatment, and it can also inhibit HBV replication through ISGs and some epigenetic modification occurred during its treatment. HBV is a DNA virus whose infection is tightly linked to its cccDNA. Epigenetic changes such as histone acetylation, DNA methylation, m6A modification, and miRNA expression is involved in its infection process. Therefore, it is important to understand the epigenetic changes involved in the pathogenesis and treatment of CHB which may provide a new therapeutic target to develop new treatment methods.

## Author contributions

ZY and DW wrote the manuscript; ZY, BS, JX, HW, HL, LC, MH, SK, and WL collected the references and drew the figures. All authors contributed to the article and approved the submitted version.

## Funding

This work was supported by the Fundamental Research Funds of the Jilin Province, Department of Finance (Grant No. jcsz202189313), the Jilin Scientific and Technological Development Program (Grant Nos. 81602377), the Changchun Scientific and Technological Development Program (Grant Nos. 21ZY26), the Jilin Province Scientific and Technological Development Program (Grant Nos. 20200801077GH) and the Jilin Province Scientific and Technological Development Program (Grant Nos. 20220505033ZP).

## Conflict of interest

The authors declare that the research was conducted in the absence of any commercial or financial relationships that could be construed as a potential conflict of interest.

The handling editor declared a shared parent affiliation with the authors at the time of review.

## Publisher’s note

All claims expressed in this article are solely those of the authors and do not necessarily represent those of their affiliated organizations, or those of the publisher, the editors and the reviewers. Any product that may be evaluated in this article, or claim that may be made by its manufacturer, is not guaranteed or endorsed by the publisher.
